# Clinical factors associated with CT-confirmed pneumonia in febrile patients: a retrospective study addressing verification bias with inverse probability weighting

**DOI:** 10.1186/s12879-026-14083-y

**Published:** 2026-07-24

**Authors:** Shu-Rou Qin, Xin-Qi Li, Jing-Qi Pei, Lin-Ni Tang, Hong Cai, Ying-Gang Zhu, Shao-Hua Cui

**Affiliations:** Department of Pulmonary and Critical Care Medicine, Huadong Hospital, Fudan University, Shanghai, 200040 China

**Keywords:** Febrile patients, Pneumonia, Verification bias, LASSO regression, Inverse probability weighting

## Abstract

**Background:**

Identifying pneumonia among febrile patients is clinically important, particularly in real-world settings where chest computed tomography (CT) is not performed uniformly. We aimed to identify clinical factors associated with CT-confirmed pneumonia in febrile patients and to examine the robustness of these associations after accounting for potential verification bias due to non-random CT completion.

**Methods:**

This retrospective observational study included 902 febrile patients from Huadong Hospital between December 2022 and May 2023. The primary analysis was restricted to patients who underwent chest CT, among whom CT findings were categorized as no pneumonia (*n* = 335) or pneumonia (*n* = 210); 357 did not undergo CT. Baseline characteristics were compared using the Wilcoxon rank-sum test or chi-square test, as appropriate. Right-skewed biomarkers, including C-reactive protein (CRP), neutrophil-to-lymphocyte ratio (NLR), and lymphocyte-to-monocyte ratio (LMR), were transformed using log(x + 1). Candidate predictors were screened using univariable logistic regression and 10-fold cross-validated least absolute shrinkage and selection operator (LASSO) regression, followed by a non-penalized multivariable logistic regression model to estimate adjusted odds ratios (ORs) and 95% confidence intervals (CIs). Model performance was evaluated by discrimination, calibration, decision curve analysis, and bootstrap internal validation (B = 1000). To address potential verification bias arising from selective CT completion, stabilized inverse probability weighting (IPW) was applied as a sensitivity analysis. Supplementary analyses evaluated the incremental discriminative value of laboratory biomarkers beyond respiratory symptoms and assessed sensitivity to a stricter pneumonia outcome definition.

**Results:**

Among patients who underwent CT, those with CT-confirmed pneumonia were older and had higher CRP levels than those without pneumonia. The final multivariable model included age, sex, body temperature, monocyte percentage, eosinophil percentage, log-transformed CRP, and log-transformed LMR. Older age (OR 1.02, 95% CI 1.01–1.03, *p* < 0.001) and higher log-transformed CRP (OR 1.42, 95% CI 1.18–1.70, *p* < 0.001) were robustly associated with CT-confirmed pneumonia. A positive association was also observed for higher log-transformed LMR (OR 1.86, 95% CI 1.10–3.15, *p* = 0.021), but this association was sensitive to parameterization and should be regarded as exploratory. The model showed modest discrimination, with an apparent area under the receiver operating characteristic curve (AUC) of 0.691 and a bootstrap-corrected AUC of 0.673 (95% CI 0.630–0.716); the Brier score was 0.211. The final model outperformed CRP, NLR, and white blood cell count alone in discrimination. Respiratory symptoms were nearly identically distributed between patients with and without pneumonia and did not improve discrimination beyond demographics, whereas adding laboratory biomarkers significantly improved the AUC from 0.644 to 0.697 (*p* = 0.003). In IPW-adjusted analyses, the estimates for age and log-transformed CRP remained materially unchanged. Sensitivity analyses showed that the exploratory association involving LMR was dependent on parameterization, remaining directionally consistent in log-transformed and categorized models but not significant when modeled as a raw continuous variable.; a stricter outcome definition requiring compatible respiratory symptoms or hypoxemia yielded directionally consistent estimates for the main predictors.

**Conclusions:**

Older age and higher inflammatory burden, particularly elevated CRP, were independently associated with CT-confirmed pneumonia in febrile patients. Adjustment for verification bias using IPW supported the robustness of the main findings. A multivariable model based on these factors showed modest discrimination and may assist with preliminary risk stratification, whereas the positive association involving LMR was parameterization-dependent and should be interpreted as an exploratory finding.

**Supplementary Information:**

The online version contains supplementary material available at https://doi.org/10.1186/s12879-026-14083-y.

## Introduction

Pneumonia remains a major cause of morbidity and mortality worldwide, particularly among older adults and patients presenting with febrile illness [1, 2]. Timely and accurate diagnosis is essential for guiding appropriate management and improving outcomes, yet distinguishing pneumonia from other causes of fever remains challenging in routine clinical practice [3, 4]. Symptoms, vital signs, and routine laboratory findings often lack sufficient specificity when used in isolation, leading to diagnostic uncertainty and potential delays in treatment [4–6].

Chest computed tomography (CT) can help identify pulmonary infiltrates and served as the imaging reference in this study [3, 7]. However, CT is not performed uniformly across all febrile patients because of cost, radiation exposure, limited availability, and variations in clinical judgment [3, 7]. Consequently, clinicians often rely on combinations of clinical features and inflammatory biomarkers to identify patients who may benefit from further imaging evaluation [3–7].

Several clinical prediction models have been developed to aid pneumonia identification in different settings. Simplified tools such as the DRC model (dyspnea, respiratory rate > 20/min, and C-reactive protein > 20 mg/L) demonstrated good discrimination for pneumonia prediction in patients with acute febrile respiratory illness in primary care settings [3]. Other models incorporating clinical signs, symptoms, and C-reactive protein (CRP) have also shown useful performance in both adult and pediatric populations [5, 6, 8, 9]. Machine-learning approaches applied to electronic health records or multimodal clinical data have likewise shown promise for pneumonia risk prediction, although their performance may vary across settings and populations [7, 10].

Among candidate biomarkers, CRP has consistently demonstrated value in supporting pneumonia diagnosis across different clinical settings [3, 5, 6]. Inflammatory ratios derived from routine complete blood counts, particularly the neutrophil-to-lymphocyte ratio (NLR) and lymphocyte-to-monocyte ratio (LMR), have also been investigated as accessible markers of systemic inflammation and infection [11–14]. NLR has shown prognostic value in community-acquired pneumonia, correlating with disease severity and adverse outcomes [12, 13], whereas LMR has demonstrated diagnostic utility in differentiating pneumonia from upper respiratory tract infection in children [14]. However, the role of these biomarkers in real-world febrile adult populations remains uncertain [11].

A critical methodological issue in studies evaluating pneumonia predictors is verification bias, also known as work-up bias, which arises when the reference standard, such as chest CT, is not applied uniformly across the study population [15–17]. If CT completion is influenced by patient characteristics, such as older age, higher inflammatory burden, or clinician suspicion, estimates of association between candidate predictors and pneumonia may be biased [15, 16]. Analyses restricted to patients who undergo the reference test may therefore yield misleading results. Inverse probability weighting (IPW) and related methods provide statistical approaches to address such bias by weighting observations according to the probability of receiving the reference test, thereby reconstructing a pseudo-population in which verification is less dependent on observed covariates [17, 18].

Despite increasing recognition of verification bias in diagnostic research, few studies on pneumonia prediction have explicitly addressed this issue [15–17]. Moreover, models that simultaneously incorporate demographic characteristics, clinical features, and inflammatory biomarkers while accounting for selective CT verification remain limited. Therefore, we conducted a retrospective observational study to investigate the clinical characteristics and factors associated with CT-confirmed pneumonia in febrile patients, with particular attention to verification bias. Our specific aims were: (1) to identify independent factors associated with pneumonia using LASSO regression and multivariable logistic modeling; (2) to evaluate the performance of the resulting model; and (3) to assess the impact of verification bias using IPW as a sensitivity analysis. We hypothesized that older age and elevated inflammatory burden would be independently associated with pneumonia, and that failure to account for selective CT completion could bias these associations.

## Methods

### Study design and patient selection

**Fig. 1:**
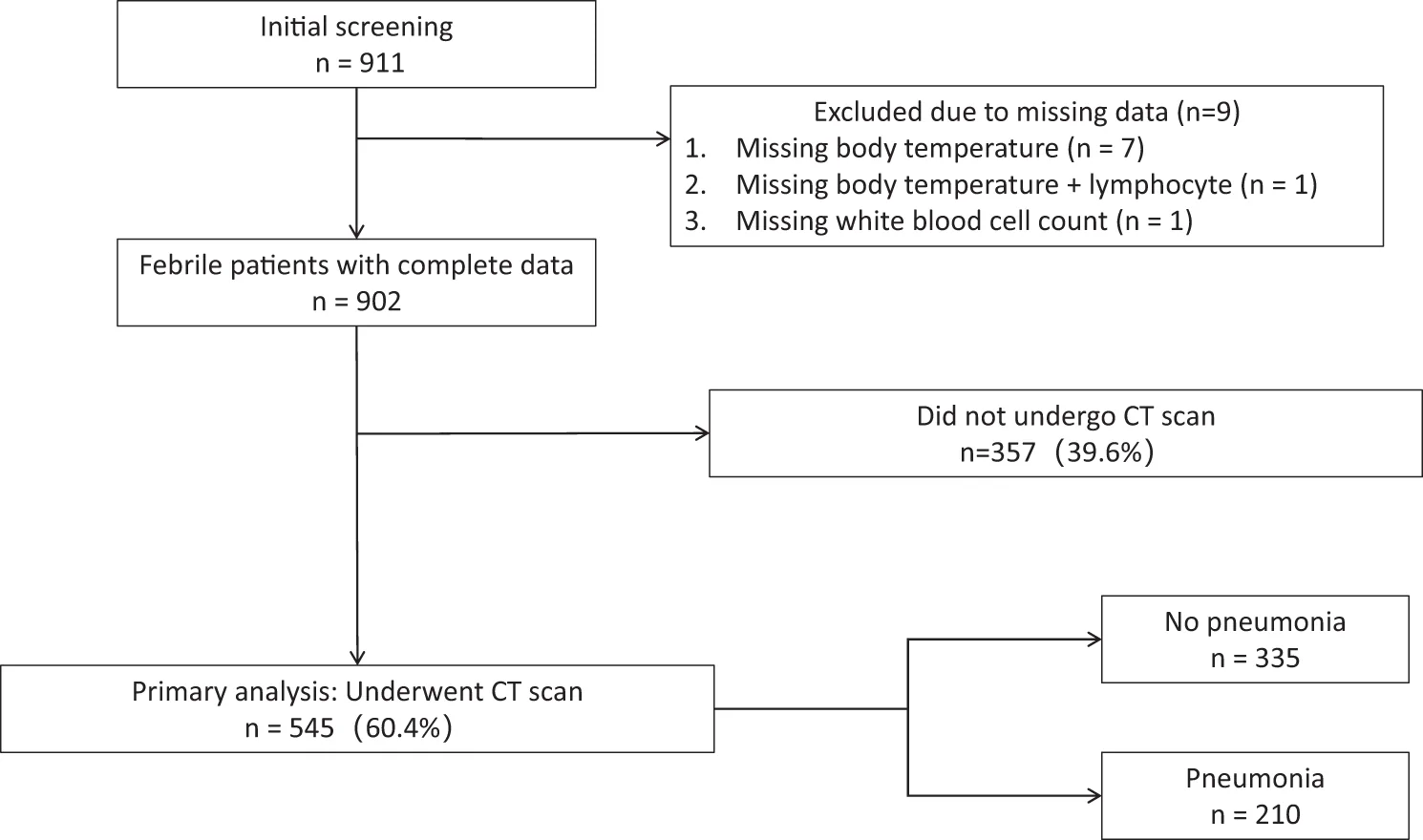
Enrollment flow diagram. A total of 902 febrile patients were included in the full dataset. Among them, 357 patients did not undergo chest CT, 335 had CT findings without pneumonia, and 210 had CT-confirmed pneumonia. The primary analysis was restricted to the 545 patients who completed CT examination, with pneumonia status defined according to CT findings

This retrospective observational study included febrile patients who presented to the fever clinic of Huadong Hospital between December 2022 and May 2023. Febrile patients were defined as those with a documented history of fever (axillary temperature > 37.3°C) within the preceding week, regardless of the body temperature measured at the clinic visit. Initially, 911 febrile patients presented to the fever clinic during the study period. No explicit age restriction was applied. However, because the fever clinic is located within an adult tertiary-care general hospital, it primarily serves an adult population. During the study period, no patient younger than 14 years was enrolled; the youngest patient was 14 years old, and only 15 patients (1.7%) were younger than 18 years. Of the 911 eligible patients, 9 (1.0%) were excluded because of missing data, including 7 with missing body temperature records, 1 with missing both body temperature and lymphocyte count, and 1 with missing white blood cell count. After these exclusions, the final analytic cohort comprised 902 patients (99.0% of the initial sample) with complete data for all core variables (demographics, vital signs, and laboratory parameters) used in the primary analyses (Fig. 1). Because the proportion of missing data was minimal and no missing values remained in the final analytic dataset, complete-case analysis was performed and no imputation procedures were applied. Missing data patterns for supplementary variables (respiratory symptoms, SpO₂, and comorbidities) are summarized in Supplementary Table S13.

The primary analytic cohort consisted of patients who underwent chest CT examination. According to CT findings, these patients were classified into a no-pneumonia group and a pneumonia group. Patients who did not undergo CT were excluded from the primary etiologic analysis but were retained for assessment of CT-selection bias. This study was conducted in accordance with the principles of the Declaration of Helsinki. The study was approved by the Ethics Committee of Huadong Hospital, Fudan University (Approval No. 20250137), and the requirement for informed consent was waived because of the retrospective design. To clarify the clinical workflow, this model was conceptualized as an early risk-stratification tool applied at the initial encounter in the fever clinic, utilizing universally available baseline data (demographics and rapid laboratory tests). It is intended to estimate the objective probability of pneumonia prior to, and independent of, the final advanced imaging decision, rather than serving as a post-CT diagnostic tool or a rigid gatekeeper that completely replaces clinical judgment.

### Data collection and variable definitions

Data were extracted from the electronic medical records of all eligible patients. Core variables, which constituted the primary analytic dataset, comprised demographic characteristics (age and sex), vital signs (body temperature), and laboratory markers including white blood cell count (WBC), neutrophil percentage, lymphocyte percentage, monocyte percentage, eosinophil percentage, and C-reactive protein (CRP). Derived inflammatory indices—the neutrophil-to-lymphocyte ratio (NLR) and lymphocyte-to-monocyte ratio (LMR)—were calculated from the corresponding blood cell measurements. These core variables had near-complete ascertainment; only 9 of 911 patients (1.0%) had missing core-variable data and were excluded, yielding a primary analytic cohort of 902 patients with complete core data.

Supplementary variables were also extracted: documented comorbidities (hypertension, diabetes mellitus, coronary artery disease, chronic lung disease, chronic kidney disease, and malignancy), respiratory symptoms at presentation (cough, sputum production, dyspnea, hemoptysis, chest pain, sore throat, and upper respiratory symptoms [rhinorrhea, nasal congestion, or sneezing]), and pulse oximetry (SpO₂) when recorded. Because these supplementary variables were retrospectively abstracted from unstructured clinical notes, ascertainment was less complete than for the core laboratory and demographic data; no patient was excluded on the basis of missing supplementary variables. Missing data patterns for supplementary variables are summarized in Supplementary Table S13. Analyses involving these variables were restricted to patients with complete data for the specific variables required (e.g., *n* = 534 for the incremental discriminative value analysis; *n* = 544 for the strict-outcome sensitivity analysis).

All vital signs and laboratory variables were obtained from the initial fever-clinic encounter. Body temperature referred to the measurement recorded at presentation, whereas laboratory parameters were derived from the first blood sample collected during that visit. No repeat measurements, peak values, or post-treatment measurements were used in the analyses.

Chest CT findings were categorized into three groups: no CT performed, CT without pneumonia, and CT with pneumonia. During the study period, chest radiography was not available within the fever clinic. Therefore, chest CT served as the primary thoracic imaging modality when pulmonary infection was clinically suspected and was not performed as a secondary examination following chest radiography.No institutional protocol governed chest CT ordering during the study period. CT decisions were at the treating physician’s discretion and, as shown in Supplementary Table S4a, were primarily driven by older age and markers of systemic inflammation (e.g., CRP and WBC alterations). The primary outcome was CT-confirmed pneumonia, defined as the presence of new inflammatory pulmonary infiltrates on chest CT as interpreted by attending radiologists in routine clinical practice, with the final diagnosis confirmed by the treating respiratory physician based on the complete clinical record. This imaging-centered definition was adopted because chest CT served as the reference standard for pneumonia diagnosis in the fever-clinic setting, where systematic microbiologic confirmation was not available. Guideline-based pneumonia definitions typically require compatible respiratory symptoms in addition to radiographic findings; however, respiratory symptoms are highly prevalent among febrile patients regardless of pneumonia status (Figure S6), limiting their standalone discriminatory value. To evaluate whether reliance on an imaging-based definition influenced the results, a sensitivity analysis was performed using a stricter outcome definition that additionally required at least one compatible respiratory symptom (cough, sputum production, or dyspnea) or SpO₂ <95%; for patients without documented SpO₂ (59.8% of the CT-complete cohort), the SpO₂ criterion was conservatively considered unmet.

Because CRP, NLR, and LMR showed right-skewed distributions, these biomarkers were transformed using log(x + 1). The distributional characteristics of these markers before and after transformation are summarized in Table S1. For sensitivity analyses, z-standardized values of the log-transformed biomarkers were additionally generated. For clinically interpretable analyses, categorical variables were created using predefined cutoffs as follows: age (<65, 65–74, ≥75 years), body temperature (<37.3, 37.3-<38.0, ≥38.0 °C), WBC (<4, 4–10, >10 × 10^9/L), CRP (<10, 10–50, ≥50 mg/L), and NLR (<3, 3–5, ≥5).

### Statistical analysis

All statistical analyses were performed using R version 4.5.0. A two-sided *p* value < 0.05 was considered statistically significant.

Continuous variables were summarized as median with interquartile range (IQR), and categorical variables as number with percentage. Baseline differences between patients with and without CT-confirmed pneumonia in the CT-complete cohort were compared using the Wilcoxon rank-sum test for continuous variables and the chi-square test for categorical variables. To assess potential verification bias, baseline characteristics were also compared between patients who underwent CT and those who did not.

Candidate factors associated with CT-confirmed pneumonia were first examined using univariable logistic regression in the CT-complete cohort. Thereafter, least absolute shrinkage and selection operator (LASSO) regression with 10-fold cross-validation was applied to a candidate set including demographic variables, routine laboratory parameters, and both raw and log-transformed forms of right-skewed biomarkers (CRP, NLR, and LMR). If both the raw and transformed forms of the same biomarker were retained, the log-transformed form was preferentially selected because of its more appropriate distributional properties. Variables retained by LASSO, together with clinically relevant variables, were entered into a non-penalized multivariable logistic regression model to estimate adjusted odds ratios (ORs) and 95% confidence intervals (CIs). Multicollinearity was assessed using variance inflation factors (VIFs), with VIF > 5 indicating potential concern.

SpO₂was not included as a candidate predictor in the primary model because of its high rate of missingness (59.8% in the CT-complete cohort; Supplementary Table S13). It was instead incorporated into the stricter-outcome sensitivity analysis, in which patients without documented SpO₂ were conservatively treated as not meeting the hypoxemia criterion.

As a secondary analysis, the performance of the final multivariable model was evaluated in terms of discrimination, calibration, and potential clinical usefulness. Discrimination was assessed using the area under the receiver operating characteristic curve (AUC). Internal validation was performed using bootstrap resampling (B = 1000) to estimate optimism-corrected AUC. Calibration was assessed using calibration plots, and overall predictive accuracy was summarized by the Brier score. The discrimination of the final model was further compared with CRP, NLR, and WBC alone using ROC analysis and DeLong’s test. Net reclassification improvement (NRI) and integrated discrimination improvement (IDI) were calculated as continuous (category-free) measures. To assess the precision of these estimates and to derive *p* values, 2,000 bootstrap resamples were performed, and 95% confidence intervals were obtained from the bootstrap distribution. Decision curve analysis (DCA) was used to explore potential clinical utility. In addition, patients were stratified into low-, intermediate-, and high-risk groups according to tertiles of predicted risk, and the corresponding event rates and diagnostic performance metrics were summarized. A nomogram was generated from the final multivariable model for visualization.

Because chest CT was not performed uniformly across the study population, verification bias was considered likely. To address this issue, a multivariable logistic regression model was constructed to estimate each patient’s probability of receiving a CT scan (propensity score), with age, sex, body temperature, C-reactive protein, neutrophil-to-lymphocyte ratio, white blood cell count, neutrophil percentage, lymphocyte percentage, monocyte percentage, and eosinophil percentage as covariates (Supplementary Table S4a). Stabilized inverse probability weights (sIPW) were then calculated as ${{P{\rm{(}}CT{\rm{)}}} \over {P{\rm{(}}CT\mid X{\rm{)}}}}$ for patients who underwent CT and ${{{\rm{1 - }}P{\rm{(}}CT{\rm{)}}} \over {{\rm{1 - }}P{\rm{(}}CT\mid X{\rm{)}}}}$ for those who did not, where P(CT) = 60.4% is the marginal probability of CT completion and P(CT | X) is the individual predicted probability from the propensity score model. The distribution of the stabilized weights was examined to confirm appropriate model specification (mean 1.01, range 0.62–4.32; Supplementary Table S4b). Covariate balance before and after weighting was assessed using standardized mean differences (SMDs), with absolute SMD < 0.1 considered indicative of adequate balance (Supplementary Table S4c). These weights were applied in two ways. First, a weighted logistic regression model using the CT-completion model covariates was fitted within the CT-complete cohort (quasibinomial family) and compared with the corresponding unweighted model to assess the extent of verification bias. Second, the stabilized weights were applied to the final multivariable model as a sensitivity analysis to examine whether the main associations remained robust after accounting for selective CT completion.

Several sensitivity analyses were performed: (1) a model using clinically categorized variables; (2) alternative specifications of LMR, in which log-transformed LMR was replaced by raw LMR, z-standardized log-transformed LMR, and tertile-based LMR; (3) a model excluding the LMR term; (4) interaction models including age × log-transformed NLR and age × log-transformed LMR; (5) an IPW-weighted version of the final multivariable model; and (6) a sensitivity analysis using the stricter outcome definition requiring both CT-confirmed infiltrates and at least one respiratory symptom (cough, sputum production, or dyspnea) or SpO₂ <95%. In addition, to assess the incremental discriminative value of laboratory biomarkers beyond clinical and symptom-based assessment, nested logistic regression models were compared: demographics only (Model 1: age, sex, body temperature), demographics plus respiratory symptoms (Model 2: Model 1 plus cough, sputum, dyspnea), and demographics plus symptoms plus all laboratory biomarkers from the final model (Model 3: Model 2 plus monocyte percentage, eosinophil percentage, log-transformed CRP, and log-transformed LMR). Discrimination was compared using ROC analysis with DeLong’s test, and reclassification was assessed using continuous NRI and IDI with bootstrap-based inference (1,000 resamples).

## Results

### Study population and CT verification bias

A total of 902 febrile patients were included in the full dataset, of whom 357 did not undergo chest CT, 335 had CT findings without pneumonia, and 210 had CT-confirmed pneumonia. Accordingly, 545 patients (60.4% of the full dataset) who completed CT were included in the primary analysis (Fig. 1). Patients who underwent CT differed substantially from those who did not, particularly with respect to age and several inflammatory markers. Compared with patients without CT, those who underwent CT were older (median age, 64.0 vs 39.0 years; *p* < 0.001), had lower white blood cell counts (6.0 vs 6.5 × 10^9/L; *p* < 0.001), lower neutrophil-to-lymphocyte ratios (3.69 vs 4.81; *p* < 0.001), and higher C-reactive protein levels (9.3 vs 8.2 mg/L; *p* = 0.023), supporting the presence of non-random CT verification (Table 1; Figure S2).

**Table 1:** Baseline characteristics of the study population stratified by CT completion status

Characteristic	Total (N = 902)	CT completed (n = 545)	CT not completed (n = 357)	p-value
Demographics				
Age, years	57.0 (35.0–69.0)	64.0 (42.0–73.0)	39.0 (29.0–60.0)	<0.001
Male, n (%)	380 (42.1)	226 (41.5)	154 (43.1)	0.669
Vital sign				
Body temperature, °C	37.8 (37.3–38.4)	37.8 (37.3–38.3)	37.9 (37.4–38.5)	0.048
Laboratory parameters				
White blood cell count, ×10^9^ /L	6.2 (4.9–7.9)	6.0 (4.8–7.7)	6.5 (5.3–8.4)	<0.001
Neutrophil, %	72.2 (62.8–79.2)	70.1 (60.9–78.1)	74.1 (66.3–80.1)	<0.001
Lymphocyte, %	17.8 (11.5–25.4)	19.1 (12.5–27.3)	15.5 (10.4–22.4)	<0.001
Monocyte, %	8.6 (6.7–10.6)	8.5 (6.5–10.6)	8.6 (6.9–10.9)	0.682
Eosinophil, %	0.8 (0.4–1.5)	0.8 (0.4–1.5)	0.8 (0.4–1.4)	0.663
C-reactive protein, mg/L	8.9 (4.3–17.9)	9.3 (4.7–21.2)	8.2 (4.1–15.2)	0.023
Neutrophil-to-lymphocyte ratio	4.11 (2.48–6.88)	3.69 (2.23–6.20)	4.81 (3.01–7.62)	<0.001
Lymphocyte-to-monocyte ratio	2.00 (1.41–2.81)	2.16 (1.48–3.10)	1.84 (1.31–2.47)	<0.001

*Data are presented as median (interquartile range) or number (%). p-values were calculated using the Wilcoxon rank-sum test for continuous variables and the chi-square test for categorical variables, comparing patients who underwent CT with those who did not*

### Baseline characteristics within the CT-complete cohort

Within the CT-complete cohort, patients with pneumonia were older than those without pneumonia (median age, 66.5 vs 61.0 years; *p* < 0.001), more likely to be male (48.6% vs 37.0%; *p* = 0.010), and had higher CRP levels (11.9 vs 8.0 mg/L; *p* < 0.001). In addition, monocyte percentage was lower (8.0% vs 8.8%; *p* = 0.0045), eosinophil percentage was lower (0.7% vs 0.8%; *p* = 0.039), and lymphocyte-to-monocyte ratio was higher (2.43 vs 2.02; *p* = 0.002) in the pneumonia group, whereas body temperature, white blood cell count, neutrophil percentage, lymphocyte percentage, and neutrophil-to-lymphocyte ratio did not differ significantly between groups (Table 2).

**Table 2:** Baseline characteristics of patients who underwent CT, stratified by pulmonary imaging findings

Characteristic	No pneumonia on CT (n = 335)	Pneumonia on CT (n = 210)	p-value
**Demographics**			
Age, years	61.0 (38.0–71.0)	66.5 (57.3–76.0)	<0.001
Male, n (%)	124 (37.0)	102 (48.6)	0.01
**Vital sign**			
Body temperature, °C	37.8 (37.4–38.4)	37.8 (37.2–38.3)	0.163
**Laboratory parameters**			
White blood cell count, ×10^9^ /L	6.1 (4.9–7.5)	6.0 (4.6–8.1)	0.688
Neutrophil, %	70.2 (61.4–78.4)	69.8 (60.3–77.8)	0.932
Lymphocyte, %	19.0 (12.2–26.7)	19.5 (13.1–28.7)	0.264
Monocyte, %	8.8 (6.8–11.2)	8.0 (6.4–9.9)	0.0045
Eosinophil, %	0.8 (0.4–1.6)	0.7 (0.3–1.3)	0.039
C-reactive protein, mg/L	8.0 (4.1–16.7)	11.9 (5.2–31.0)	<0.001
Neutrophil-to-lymphocyte ratio	3.67 (2.31–6.52)	3.70 (2.05–5.88)	0.346
Lymphocyte-to-monocyte ratio	2.02 (1.40–2.95)	2.43 (1.68–3.33)	0.002

*Data are presented as median (interquartile range) or number (%). Pneumonia was defined based on radiological findings from chest CT scans interpreted by attending radiologists. p-values were calculated using the Wilcoxon rank-sum test for continuous variables and the chi-square test for categorical variables, comparing patients with and without CT-confirmed pneumonia*

### Univariable logistic regression

In univariable logistic regression analyses, older age (OR 1.02, 95% CI 1.01–1.03; *p* < 0.001), higher CRP (OR 1.02, 95% CI 1.01–1.03; *p* < 0.001), higher log-transformed CRP (OR 1.50, 95% CI 1.26–1.78; *p* < 0.001), male sex (OR 1.61, 95% CI 1.13–2.28; *p* = 0.008), lower monocyte percentage (OR 0.93, 95% CI 0.88–0.98; *p* = 0.007), and higher log-transformed LMR (OR 1.84, 95% CI 1.23–2.74; *p* = 0.003) were associated with CT-confirmed pneumonia (Table S2). By contrast, raw continuous LMR was not significantly associated with pneumonia (OR 1.06, 95% CI 0.99–1.12; *p* = 0.092), and neither NLR nor log-transformed NLR showed significant associations (*p* = 0.274 and *p* = 0.318, respectively) (Table S2).

### LASSO variable selection

**Fig. 2:**
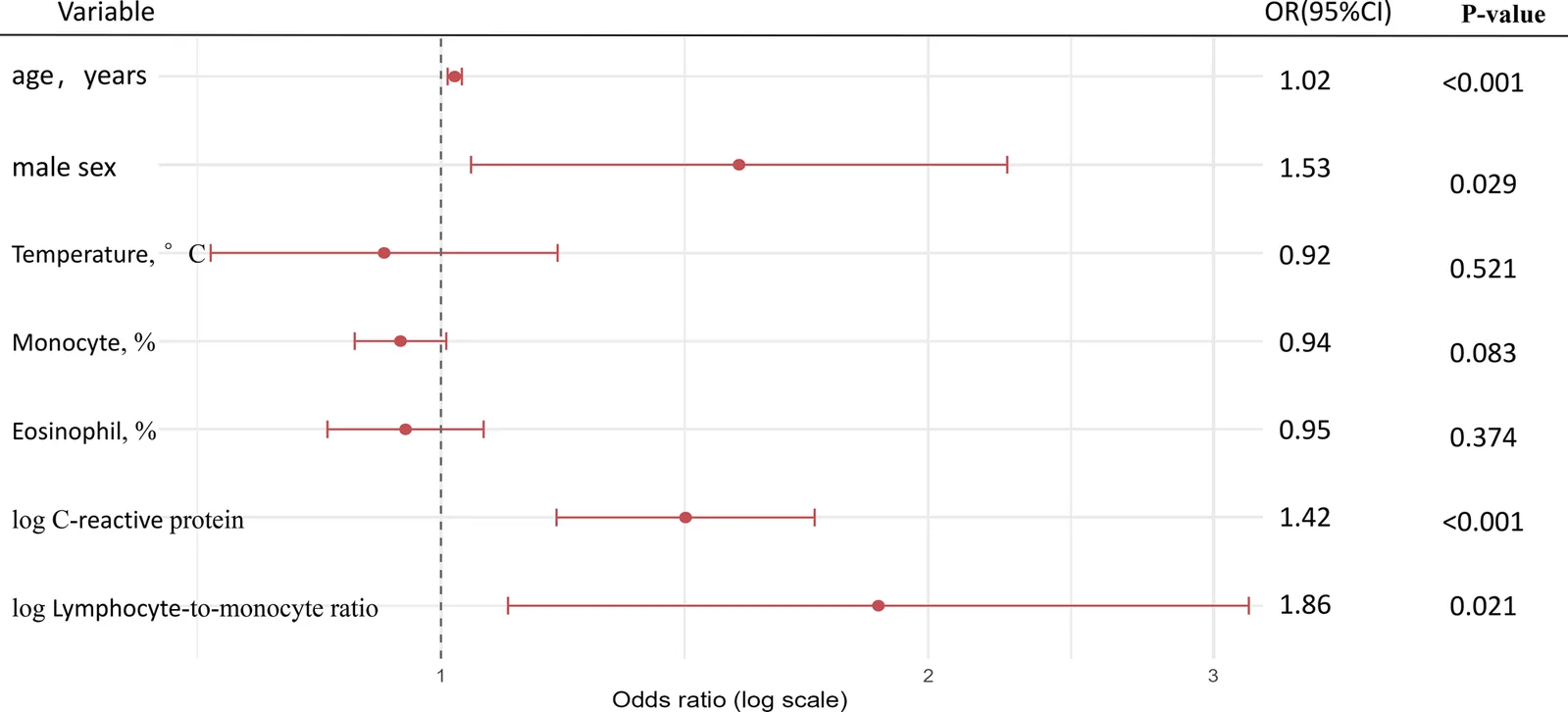
Forest plot of the final multivariable logistic regression model, the final model included age, sex, body temperature, monocyte percentage, eosinophil percentage, log-transformed C-reactive protein, and log-transformed lymphocyte-to-monocyte ratio. Points indicate odds ratios, and horizontal bars indicate 95% confidence intervals. The vertical dashed line indicates an odds ratio of 1.0

LASSO regression with 10-fold cross-validation identified eight predictors with non-zero coefficients at lambda.min (0.01416), including age, male sex, body temperature, CRP, log-transformed CRP, monocyte percentage, eosinophil percentage, and log-transformed LMR (Table S3; Figure S1). After consideration of collinearity and clinical interpretability, the final multivariable logistic regression model included age, sex, body temperature, monocyte percentage, eosinophil percentage, log-transformed CRP, and log-transformed LMR (Table 3; Fig. 2). No substantial multicollinearity was observed, with all variance inflation factors below 5.

**Table 3:** Multivariable logistic regression analysis for factors associated with pneumonia in patients who underwent CT

Variable	Odds Ratio	95% CI	p-value
Age, years	1.02	1.01–1.03	<0.001
Male sex	1.53	1.04–2.24	0.029
Body temperature, °C	0.92	0.72–1.18	0.521
Monocyte, %	0.94	0.88–1.01	0.083
Eosinophil, %	0.95	0.85–1.06	0.374
log C-reactive protein	1.42	1.18–1.70	<0.001
log Lymphocyte-to-monocyte ratio	1.86	1.10–3.15	0.021

*CRP and LMR were natural log-transformed using log(x + 1) because of right-skewed distributions. The multivariable model included all variables shown in the table. No substantial multicollinearity was observed (all variance inflation factors < 5)*

### Multivariable logistic regression model

In the final multivariable model, older age remained independently associated with CT-confirmed pneumonia (OR 1.02, 95% CI 1.01–1.03; *p* < 0.001), as did male sex (OR 1.53, 95% CI 1.04–2.24; *p* = 0.029), higher log-transformed CRP (OR 1.42, 95% CI 1.18–1.70; *p* < 0.001), and higher log-transformed LMR (OR 1.86, 95% CI 1.10–3.15; *p* = 0.021) (Table 3). Monocyte percentage (OR 0.94, 95% CI 0.88–1.01; *p* = 0.083), eosinophil percentage (OR 0.95, 95% CI 0.85–1.06; *p* = 0.374), and body temperature (OR 0.92, 95% CI 0.72–1.18; *p* = 0.521) were not statistically significant in the adjusted model (Table 3).

### Model discrimination, calibration, and reclassification

**Fig. 3:**
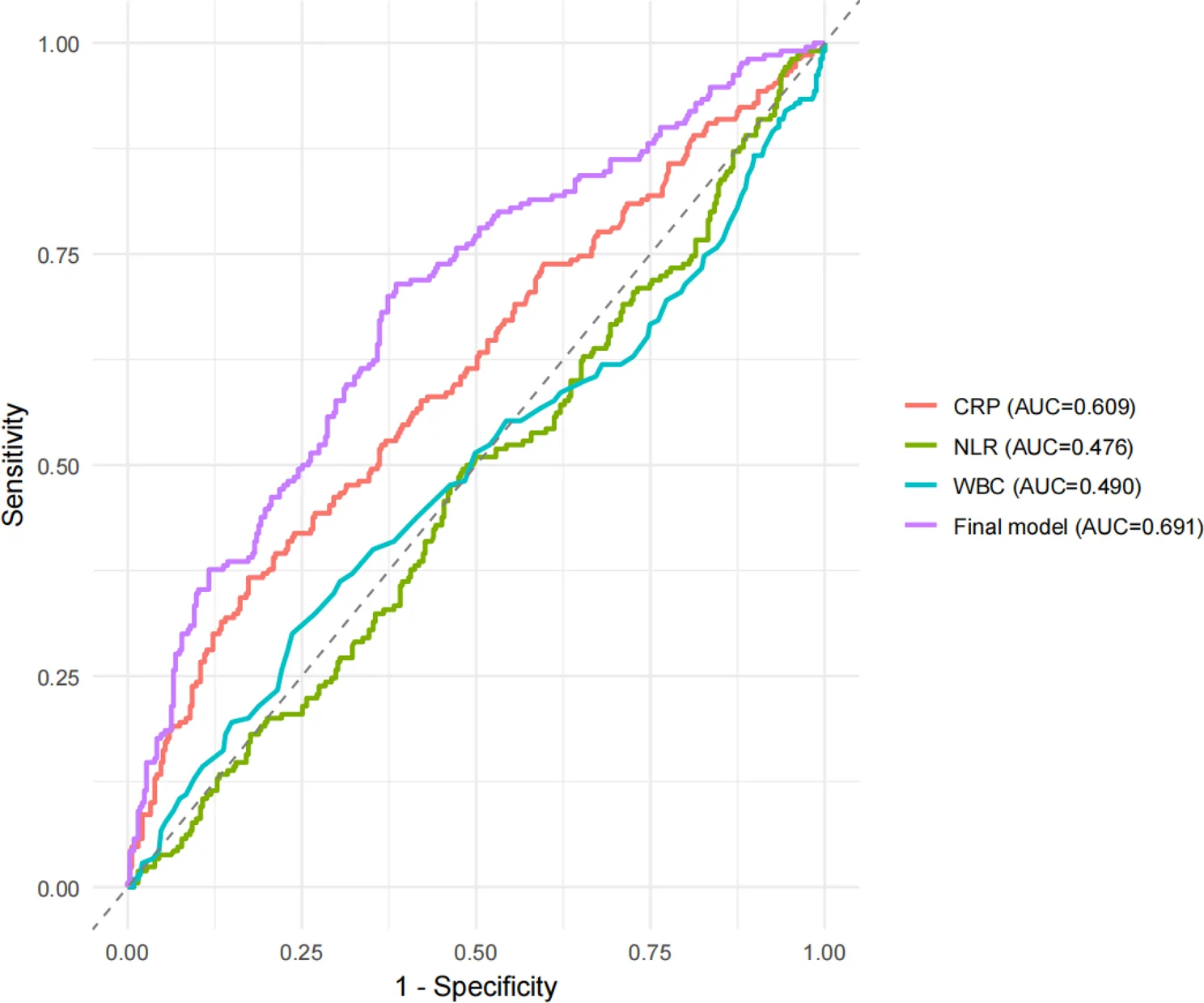
ROC curve comparison between the final model and CRP, NLR, and WBC. The final multivariable model showed better discrimination for CT-confirmed pneumonia than CRP, NLR, or WBC used as single markers. The area under the curve for the final model was 0.691. AUCs, 95% confidence intervals, optimal youden cutoffs, and DeLong test results are reported in the corresponding results table

**Fig. 4:**
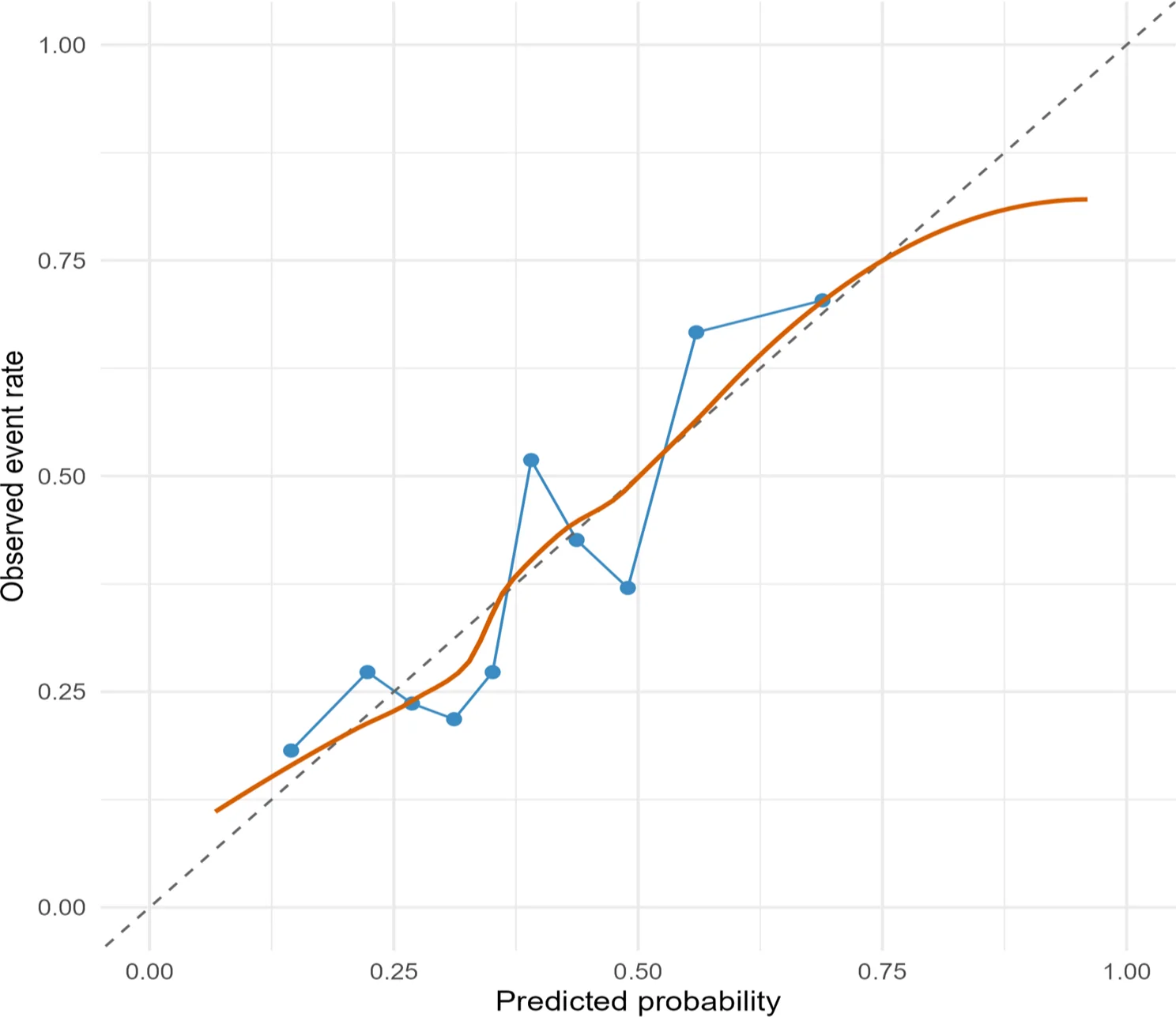
Calibration curve of the final model. The plot shows the agreement between predicted probabilities and observed event rates for CT-confirmed pneumonia. The dashed diagonal line represents perfect calibration. The blue points and line represent calibration by deciles of predicted risk. The orange smooth curve shows the nonparametric loess-calibrated relationship between predicted probabilities and observed event rates

The final model showed modest discrimination, with an apparent AUC of 0.691 (95% CI 0.645–0.736), which decreased to a bootstrap-corrected AUC of 0.673 (95% CI 0.630–0.716) after internal validation; the optimism was 0.018 and the Brier score was 0.211 (Table S9). Calibration analysis suggested acceptable agreement between predicted and observed risks (Fig. 4). In ROC comparisons, the final model outperformed CRP alone (AUC 0.609, 95% CI 0.560–0.659; DeLong *p* < 0.001), NLR alone (AUC 0.476, 95% CI 0.426–0.526; DeLong *p* < 0.001), and white blood cell count alone (AUC 0.490, 95% CI 0.438–0.541; DeLong *p* < 0.001) (Fig. 3; Table S7). Reclassification analyses further supported the final model over single-marker comparators, with total NRI of 0.418 (95% CI 0.226–0.639; *p* < 0.001) versus CRP, 0.568 (95% CI 0.381–0.755; *p* < 0.001) versus NLR, and 0.538 (95% CI 0.383–0.746; *p* < 0.001) versus WBC. The corresponding IDI values were 0.063 (*p* = 0.001), 0.105 (*p* < 0.001), and 0.102 (*p* < 0.001), respectively (Table S8).

### Decision curve analysis and risk stratification

**Fig. 5:**
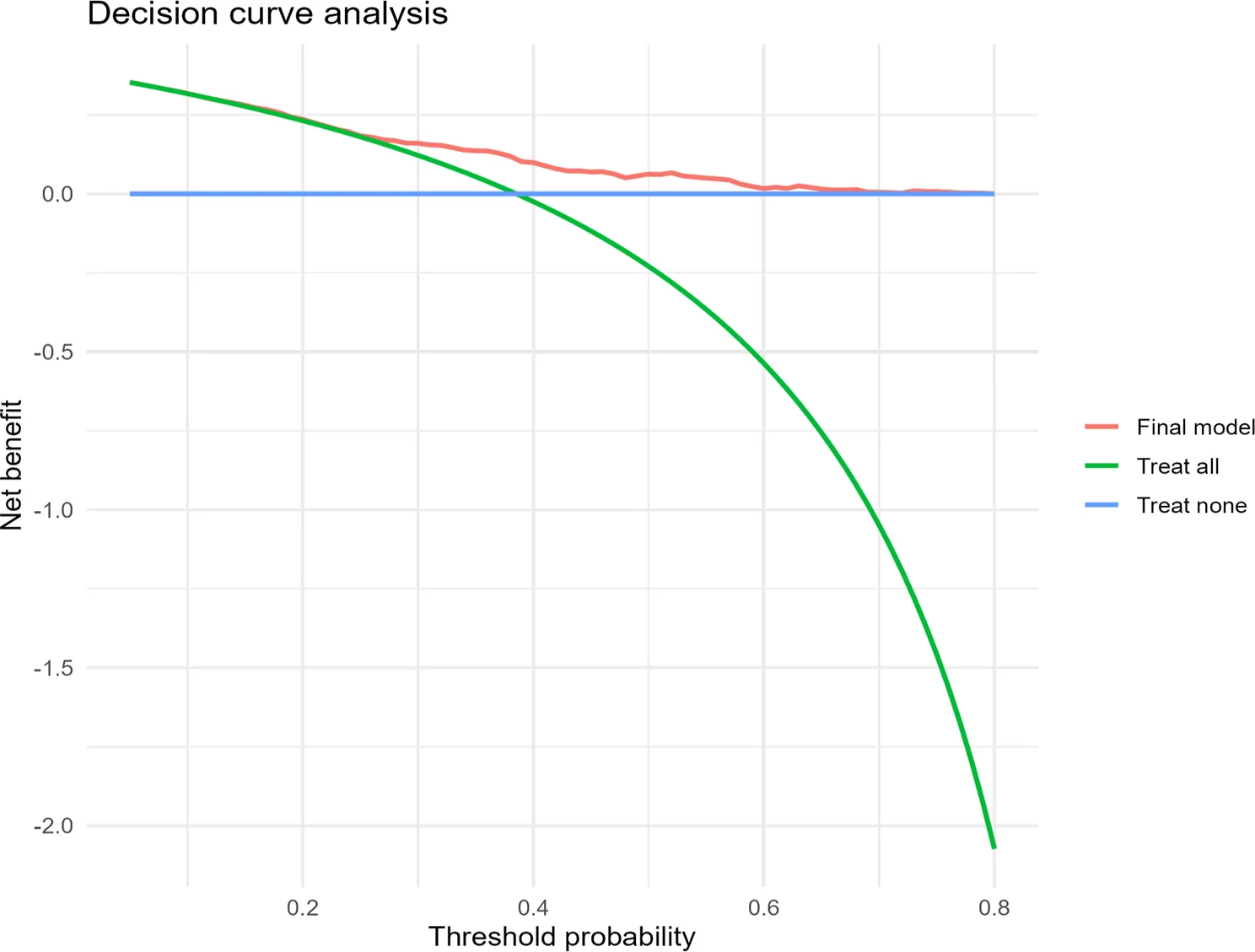
Decision curve analysis. Net benefit was plotted across a range of threshold probabilities and compared with the treat-all and treat-none strategies. The final model provided greater net benefit than default strategies over a clinically relevant range of threshold probabilities

Decision curve analysis indicated that the final model provided greater net benefit than treat-all or treat-none strategies across a clinically relevant range of threshold probabilities (Fig. 5). Risk stratification based on tertiles of predicted probability identified progressively increasing event rates from 22.0% in the low-risk group to 37.6% in the intermediate-risk group and 56.0% in the high-risk group (Table S6). When the high-risk group was used as the positive threshold, sensitivity, specificity, positive predictive value, negative predictive value, and accuracy were 48.6%, 76.1%, 56.0%, 70.2%, and 65.5%, respectively. When the intermediate- plus high-risk groups were combined, the corresponding values were 81.0%, 42.4%, 46.8%, 78.0%, and 57.2% (Table S6). A nomogram based on the final model was generated to facilitate individualized risk estimation (Figure S5).

### Respiratory symptoms, SpO₂, and comorbidities

Respiratory symptoms were documented in 534 of 545 (98.0%) patients in the CT-complete cohort. At least one respiratory symptom (cough, sputum production, or dyspnea) was present in 75.1% of patients with pneumonia and 75.4% of those without (*p* = 1.000, Fisher’s exact test; Figure S6; Table S10). Dyspnea was the only individual symptom more frequent in the pneumonia group (7.7% vs 3.4%, *p* = 0.046), though its prevalence was low and was not accompanied by a significant difference in SpO₂ (SpO₂ <95%: 10.5% vs 8.8%, *p* = 0.843; Table S10). SpO₂data were available for only 219 of 545 patients (40.2%, Supplementary Table S13). Regarding comorbidities, patients with pneumonia had a higher prevalence of hypertension (29.7% vs 20.6%, *p* = 0.023) and malignancy (7.2% vs 2.8%, *p* = 0.029) than those without pneumonia (Table S10).

### Incremental discriminative value of laboratory biomarkers

In the incremental discriminative value analysis (*n* = 534 with complete symptom and laboratory data), adding respiratory symptoms to a demographics-only model did not significantly improve the AUC (Model 1: 0.638 vs Model 2: 0.644; DeLong *p* = 0.415), with a non-significant NRI of 0.069 (95% CI −0.031 to 0.174, *p* = 0.184) and IDI of 0.007 (*p* = 0.076). By contrast, adding all four laboratory biomarkers to the symptom-based model significantly improved the AUC from 0.644 to 0.697 (DeLong *p* = 0.003; NRI 0.369, 95% CI 0.208–0.535, *p* < 0.001; IDI 0.057, 95% CI 0.038–0.078, *p* < 0.001; Figure S8 and Figure S9; Table S12). Notably, laboratory biomarkers alone without symptoms (Model 3b) achieved comparable discrimination (AUC 0.695) and significantly outperformed the symptom-based model (Model 3b vs Model 2, DeLong *p* = 0.009).

### Stricter outcome definition

In the sensitivity analysis applying the stricter outcome definition (CT-confirmed infiltrates plus respiratory symptoms or SpO₂<95%; 161 events vs 210 under the primary definition, *n* = 544), effect estimates remained directionally consistent for all variables. Age (OR 1.01 vs 1.02), log-transformed CRP (OR 1.36 vs 1.42), and log-transformed LMR (OR 1.98 vs 1.84) all retained statistical significance; male sex was attenuated to borderline significance (OR 1.46, 95% CI 0.98–2.18, *p* = 0.060; Table S11; Figure S7). The AUC under the stricter definition was 0.668, compared with 0.693 under the primary definition.

### Other sensitivity analyses

Sensitivity analyses showed that the main findings for age and CRP were robust across alternative model specifications (Table S5A; Figure S4). In the clinically categorized model, age 65–74 years (OR 1.67, 95% CI 1.08–2.60; *p* = 0.022), age ≥ 75 years (OR 2.07, 95% CI 1.31–3.27; *p* = 0.002), and CRP ≥ 50 mg/L (OR 3.90, 95% CI 2.00–7.62; *p* < 0.001) were associated with pneumonia. The association involving LMR depended on parameterization: it remained significant when modeled as log-transformed LMR (OR 1.86, 95% CI 1.10–3.15; *p* = 0.021), z-standardized log-transformed LMR (OR 1.31, 95% CI 1.04–1.64; *p* = 0.021), and the highest tertile of LMR (OR 1.93, 95% CI 1.12–3.32; *p* = 0.017), but not when modeled as a raw continuous variable (OR 1.04, 95% CI 0.97–1.11; *p* = 0.277) (Table S5A-B). After inverse probability weighting, all covariate standardized mean differences were reduced to below 0.04, confirming acceptable balance between patients who did and did not undergo CT (Supplementary Table S4c). In the IPW-weighted version of the final model, effect estimates for age (OR 1.02, 95% CI 1.01–1.03; *p* < 0.001) and log-transformed CRP (OR 1.42, 95% CI 1.17–1.73; *p* < 0.001) remained materially unchanged, while log-transformed LMR also retained a positive association (OR 1.99, 95% CI 1.13–3.51; *p* = 0.017) (Table S5A-B). No significant interactions were identified for age ×log-transformed LMR (likelihood ratio test *p* = 0.435) or age ×log-transformed NLR (likelihood ratio test *p* = 0.211) (Table S5B). In a supplementary IPW comparison model using age, sex, body temperature, log-transformed CRP, log-transformed NLR, and white blood cell count, weighted and unweighted estimates were also broadly similar, further supporting limited distortion of the primary findings by selective CT completion (Table S4b; Figure S3).

## Discussion

### Main findings

Our study identified older age, male sex, higher log-transformed CRP, and higher log-transformed LMR as factors independently associated with CT-confirmed pneumonia among febrile patients. The final multivariable model showed modest discrimination (optimism-corrected AUC 0.673) and acceptable calibration.Although a discrimination of this magnitude is modest, it must be interpreted within the context of a highly heterogeneous, real-world fever clinic population where symptoms heavily overlap. Within this challenging clinical scenario, the model significantly outperformed traditional clinical symptoms and single biomarkers (such as WBC, NLR, or CRP alone), and decision curve analysis (DCA) demonstrated a clear net clinical benefit across a wide range of threshold probabilities. More importantly, effect estimates remained materially similar after inverse probability weighting for selective CT completion, suggesting that the main associations were reasonably robust to verification bias.

### Comparison with previous studies

Older age is a well-recognized risk factor for pneumonia, particularly in populations with greater comorbidity burden and physiologic vulnerability [2]. Male sex has also been associated with a higher burden of pneumonia in epidemiologic studies, although the mechanisms are likely multifactorial and may involve behavioral, clinical, and immunologic differences [1, 19]. The independent association between CRP and CT-confirmed pneumonia in our cohort is consistent with prior work showing that CRP adds diagnostic information beyond symptoms and signs in patients with suspected respiratory infection [3–6]. Taken together, these findings support the clinical relevance of age and inflammatory burden as core correlates of CT-confirmed pneumonia in febrile patients.

### Limited value of respiratory symptoms

Although respiratory symptoms are central to guideline-based definitions of pneumonia, our findings suggest that in a febrile outpatient population presenting to a fever clinic, commonly assessed respiratory symptoms (cough, sputum production, and dyspnea) alone have limited capacity to distinguish patients with CT-confirmed pneumonia from those without. This likely reflects the etiologic heterogeneity of febrile illnesses in this setting, where both CT-confirmed pneumonia and alternative causes (e.g., upper respiratory infections, non-respiratory infections, or non-infectious conditions) frequently present with overlapping respiratory manifestations [20]. The incremental value analysis confirmed that laboratory biomarkers added discriminative information beyond symptoms and demographics, whereas symptoms added negligible value beyond demographics alone. These findings support the role of inflammatory biomarkers as objective adjuncts to—rather than replacements for—clinical assessment, and they provide context for the imaging-based primary outcome definition used in this study. The sensitivity analysis using the stricter outcome definition yielded directionally consistent estimates for age, CRP, and LMR, suggesting that the main associations were not artifacts of the outcome definition, although the attenuation of the association for male sex indicates some sensitivity to how pneumonia is defined.

### Cautious interpretation of LMR

By contrast, the association involving LMR should be interpreted cautiously. In our sensitivity analyses, the direction of association remained broadly consistent when LMR was log-transformed or categorized, but the raw continuous form was not significant. This pattern suggests that the relationship may be non-linear and parameterization-dependent rather than biologically robust. Previous studies and reviews have suggested that leukocyte-derived ratios may assist infection discrimination, but the diagnostic role and directionality of LMR-related measures have been heterogeneous across populations and clinical settings [11, 14]. Accordingly, we consider the LMR finding exploratory and in need of confirmation in independent cohorts with prespecified modeling of the biomarker.

### Verification bias and inverse probability weighting

A central contribution of this study is the explicit consideration of verification bias. In real-world febrile populations, chest CT is not applied uniformly, and imaging decisions are influenced by age, inflammatory markers, and clinical judgment. Restricting analysis to CT-verified patients can therefore distort estimated associations if the verification process is non-random [15]. In our cohort, patients who underwent CT were older and had higher CRP levels than those who did not, supporting the presence of selective verification. After applying stabilized IPW, the estimates for age and CRP were materially unchanged, which strengthens confidence that these main associations were not solely artifacts of CT selection. At the same time, IPW-based correction relies on modeling assumptions and cannot fully address unmeasured determinants of test use, so some residual bias remains possible [17, 18].

### Clinical utility and target population

To address the potential clinical utility of our model, it is necessary to clearly define its target population, intended decision, and the comparator clinical pathway, while contextualizing its modest performance against existing literature. The target population comprises all eligible febrile patients (≥14 years) at their initial presentation to a fever clinic. In current clinical pathways, physicians typically rely on unstructured clinical impressions or respiratory symptoms (the comparator) to decide who should undergo a chest CT. However, our data demonstrated that these common symptoms (cough, sputum, dyspnea) are highly prevalent and almost identically distributed between patients with and without CT-confirmed pneumonia (75.1% vs 75.4%), offering negligible stand-alone discriminative value. Therefore, the intended decision of our model is not to replace CT as a gold standard, nor to act as a definitive screening gatekeeper that entirely rules out disease. Instead, it serves as an early, objective triage tool to stratify patients into low-, intermediate-, and high-risk categories before imaging decisions are finalized.

Importantly, given that the low-risk group in our cohort still carries a 22.0% event rate of CT-confirmed pneumonia, this stratification should not be used to safely dismiss patients without follow-up; rather, it informs a graduated clinical management strategy. In high-volume triage settings (such as seasonal influenza or respiratory pandemic surges) or in resource-limited primary care facilities where CT availability is severely bottlenecked, this objective probability helps clinicians prioritize the high-risk group (who exhibited a 56.0% CT-confirmed pneumonia rate) for accelerated imaging and immediate intervention. Conversely, for the low-risk group, it supports closer, structured clinical monitoring or empirical management rather than immediate, reflexive CT referral, thereby optimizing scarce radiological resources without compromising patient safety.

### Comparison with existing prediction tools

From this perspective, our findings should not be directly compared with the performance of simplified prediction tools such as the DRC model [3] or similar frameworks [4]. The study populations, clinical settings, and verification processes differed substantially across studies. In particular, our analysis was conducted in a real-world febrile cohort in which CT was performed selectively, whereas prior tools were often developed in more specific populations with different diagnostic pathways and more uniform reference-standard application [3, 4]. Therefore, differences in discrimination likely reflect not only model composition but also differences in case mix, outcome definition, and verification strategy. The main contribution of our study is not to propose a high-performing stand-alone prediction tool, but to provide a clinically interpretable analysis of factors associated with CT-confirmed pneumonia while explicitly addressing potential verification bias and providing an objective risk-prioritization framework to augment initial symptom-based clinical assessment.

### Limitations

Several limitations merit consideration. First, this was a retrospective single-center study conducted in an adult-oriented tertiary-care fever clinic, with only a very small proportion of patients younger than 18 years. Therefore, the findings may not be directly generalizable to pediatric populations or to healthcare settings with different patient case-mix. Second, although selected comorbidity data were extracted from medical records and are summarized descriptively (Table S10), comorbidity ascertainment relied on clinical documentation at the fever-clinic visit and may not comprehensively capture all chronic conditions. Smoking history was not systematically recorded. These unmeasured or incompletely measured factors—including chronic lung disease, smoking status, symptom duration, respiratory rate, and oxygen saturation—may influence both pneumonia risk and clinical decision-making regarding CT referral. Among these, the high rate of missing SpO₂ data (59.8%) is a particular concern, as it precluded the inclusion of oxygen saturation in the primary predictive model. Although IPW was used to address selective CT completion, residual confounding by unmeasured factors, including chronic lung disease, smoking status, symptom duration, respiratory rate, and oxygen saturation, cannot be excluded. Third, the model’s discrimination was only modest, indicating that important determinants of CT-confirmed pneumonia were not captured in the available dataset. Fourth, external validation was not performed, so the risk estimates and stratification performance should be regarded as preliminary. Fifth, monocyte percentage and LMR are mathematically related because LMR is derived in part from monocyte counts. Although variance inflation factors were low and sensitivity analyses excluding LMR did not materially alter the main findings, some degree of redundancy between these predictors cannot be completely excluded. Sixth, the inclusion of both raw and transformed forms of skewed biomarkers in the candidate set may increase model instability, even though we applied a prespecified rule favoring log-transformed variables when both forms were retained. Seventh, the study period coincided with the unique epidemiological context following the relaxation of COVID-19 control restrictions in China. This may have resulted in an atypical patient mix, CT-ordering behavior, and spectrum of circulating infections, potentially limiting the generalizability of the findings to other periods or settings.

### Conclusions and future directions

Despite these limitations, the study has practical and methodological implications. Clinically, the results suggest that age and CRP are the most robust correlates of CT-confirmed pneumonia in this setting and may support preliminary risk stratification among febrile patients. Methodologically, the analysis illustrates how IPW can be used to examine the impact of selective imaging verification in observational diagnostic research. Future studies should externally validate these findings, incorporate additional clinical variables such as chronic comorbidities, smoking history, symptom characteristics, and physiological measurements, and explore whether more flexible modeling approaches or machine learning methods can add value once outcome definition and verification processes are handled rigorously [7, 10].

In conclusion, older age and higher inflammatory burden, particularly elevated CRP, were independently associated with CT-confirmed pneumonia in febrile patients. Adjustment for verification bias using inverse probability weighting supported the robustness of the main findings. A multivariable model based on these factors showed modest discrimination and may assist preliminary risk stratification, whereas the association involving LMR should be regarded as exploratory.

## Electronic supplementary material

Below is the link to the electronic supplementary material.

Supplementary material 1

Supplementary material 2

## Data Availability

The datasets used and/or analysed during the current study are available from the corresponding author on reasonable request.
