# Supplementary material for: Clinical factors associated with CT-confirmed pneumonia in febrile patients: a retrospective study addressing verification bias with inverse probability weighting
Source: BMC Infect Dis. 2026 Jul 24;26:1844. doi: 10.1186/s12879-026-14083-y (PMC13685075; doi:10.1186/s12879-026-14083-y)
Supplement: Supplementary file 1 [file 12879_2026_14083_MOESM1_ESM.docx]

**Table S1. Distribution of inflammatory markers before and after transformation**

| Variable | Stage | n | Mean | SD | Median | Q1 | Q3 | Min | Max |
| --- | --- | --- | --- | --- | --- | --- | --- | --- | --- |
| **CRP, mg/L** | Original | 902 | 16.26 | 22.95 | 8.88 | 4.35 | 17.94 | 0.13 | 269.46 |
|  | log(x+1) | 902 | 2.33 | 0.98 | 2.29 | 1.68 | 2.94 | 0.12 | 5.60 |
|  | z-score¹ | 902 | 0.00 | 1.00 | -0.04 | -0.67 | 0.62 | -2.25 | 3.32 |
| **NLR** | Original | 902 | 5.38 | 4.42 | 4.11 | 2.48 | 6.88 | 0.08 | 40.35 |
|  | log(x+1) | 902 | 1.67 | 0.58 | 1.63 | 1.25 | 2.06 | 0.08 | 3.72 |
|  | z-score¹ | 902 | 0.00 | 1.00 | -0.07 | -0.74 | 0.68 | -2.76 | 3.54 |
| **LMR** | Original | 902 | 2.72 | 6.78 | 2.00 | 1.41 | 2.81 | 0.37 | 183.60 |
|  | log(x+1) | 902 | 1.15 | 0.43 | 1.10 | 0.88 | 1.34 | 0.31 | 5.22 |
|  | z-score¹ | 902 | 0.00 | 1.00 | -0.12 | -0.63 | 0.44 | -1.95 | 9.48 |

*CRP, C-reactive protein; NLR, neutrophil-to-lymphocyte ratio; LMR, lymphocyte-to-monocyte ratio; SD, standard deviation; Q1, first quartile; Q3, third quartile.*

*¹ Z-scores were calculated from log(x+1)-transformed values.*

**Table S2. Univariate logistic regression analysis for factors associated with pneumonia in patients who underwent CT**

| **Variable** | **n** | **Odds Ratio** | **95% CI** | **P-value** |
| --- | --- | --- | --- | --- |
| Age, years | 545 | 1.02 | 1.01–1.03 | <0.001 |
| log C-reactive protein¹ | 545 | 1.50 | 1.26–1.78 | <0.001 |
| C-reactive protein, mg/L | 545 | 1.02 | 1.01–1.03 | <0.001 |
| log Lymphocyte-to-monocyte ratio² | 545 | 1.84 | 1.23–2.74 | 0.003 |
| Monocyte, % | 545 | 0.93 | 0.88–0.98 | 0.007 |
| Male sex | 545 | 1.61 | 1.13–2.28 | 0.008 |
| Lymphocyte-to-monocyte ratio | 545 | 1.06 | 0.99–1.12 | 0.092 |
| White blood cell count, ×10⁹/L | 545 | 1.05 | 0.99–1.12 | 0.105 |
| Body temperature, °C | 545 | 0.86 | 0.70–1.07 | 0.174 |
| Eosinophil, % | 545 | 0.91 | 0.80–1.04 | 0.178 |
| Lymphocyte, % | 545 | 1.01 | 0.99–1.02 | 0.249 |
| Neutrophil-to-lymphocyte ratio | 545 | 0.98 | 0.94–1.02 | 0.274 |
| log Neutrophil-to-lymphocyte ratio³ | 545 | 0.86 | 0.63–1.16 | 0.318 |
| Neutrophil, % | 545 | 1.00 | 0.99–1.01 | 0.945 |

*CI, confidence interval.*

*¹ C-reactive protein was natural log-transformed [log(x+1)] due to right-skewed distribution.
² Lymphocyte-to-monocyte ratio was natural log-transformed [log(x+1)] due to right-skewed distribution.
³ Neutrophil-to-lymphocyte ratio was natural log-transformed [log(x+1)] due to right-skewed distribution.*

*Univariable logistic regression was performed separately for each variable. Odds ratios represent the change in odds of CT-confirmed pneumonia per unit increase in the predictor.*

### **Table S3. Variable selection for pneumonia prediction using LASSO regression**

| **Category** | **Parameter / Predictor** | **Value / Coefficient** |
| --- | --- | --- |
| **LASSO Model Parameters** | $\text{λ}_{\text{min}}$ (Minimum cross-validated error) | 0.01416 |
|  | $\text{λ}_{\text{1}\text{se}}$ (Most regularized model within 1 SE) | 0.04747 |
|  | Number of predictors with non-zero coefficients | 8 |
| **Selected Predictors** | log Lymphocyte-to-monocyte ratio | 0.520 |
|  | Male sex | 0.278 |
|  | log C-reactive protein | 0.126 |
|  | Age | 0.016 |
|  | C-reactive protein | 0.008 |
|  | Monocyte percentage | -0.031 |
|  | Eosinophil percentage | -0.008 |
|  | Body temperature | -0.0003 |

*LASSO, least absolute shrinkage and selection operator; SE, standard error. Variable selection was performed using LASSO regression with 10-fold cross-validation. The optimal tuning parameter was selected according to the minimum cross-validated error criterion (lambda.min). Predictors with non-zero coefficients at lambda.min were considered retained. Positive coefficients indicate a positive association with the likelihood of pneumonia, whereas negative coefficients indicate an inverse association.*

Table S4a. Logistic regression model for CT completion (propensity score model)

| **Variable** | **β** | **SE** | **OR (95% CI)** | **P value** |
| --- | --- | --- | --- | --- |
| Intercept | 1.400 | 6.840 | — | 0.838 |
| Age, years | 0.040 | 0.004 | 1.041 (1.033–1.049) | <0.001 |
| Male sex (vs Female) | −0.124 | 0.159 | 0.883 (0.646–1.207) | 0.435 |
| Body temperature, °C | 0.073 | 0.104 | 1.076 (0.877–1.319) | 0.483 |
| CRP, mg/L | 0.014 | 0.004 | 1.014 (1.005–1.023) | 0.002 |
| NLR | 0.030 | 0.028 | 1.030 (0.975–1.088) | 0.287 |
| WBC, ×10⁹/L | −0.091 | 0.034 | 0.913 (0.854–0.976) | 0.008 |
| Neutrophil, % | −0.060 | 0.057 | 0.942 (0.842–1.053) | 0.294 |
| Lymphocyte, % | −0.020 | 0.059 | 0.980 (0.873–1.100) | 0.732 |
| Monocyte, % | −0.097 | 0.062 | 0.908 (0.804–1.025) | 0.117 |
| Eosinophil, % | −0.016 | 0.073 | 0.984 (0.853–1.134) | 0.822 |

***β****: regression coefficient; SE: standard error; OR: odds ratio; CI: confidence interval.*

*AUC = 0.744 (95% CI: 0.712–0.776). Marginal probability of CT completion = 545/902 (60.4%). The model was fitted using logistic regression with all variables entered simultaneously.*

### Table S4b. Multivariable regression analysis before and after inverse probability weighting (IPW)

| **Variable** | **Unweighted Model OR**  **(95% CI)** | **P-value** | **IPW-weighted Model OR**  **(95% CI)** | **P-value** |
| --- | --- | --- | --- | --- |
| Age (years) | 1.02 (1.01–1.03) | **<0.001** | 1.02 (1.01–1.03) | **<0.001** |
| Male sex | 1.35 (0.93–1.96) | 0.116 | 1.28 (0.87–1.88) | 0.206 |
| Body temperature (°C) | 0.89 (0.70–1.13) | 0.341 | 0.91 (0.71–1.16) | 0.447 |
| $\text{log}$ C-reactive protein¹ | 1.42 (1.18–1.71) | **<0.001** | 1.42 (1.17–1.73) | **<0.001** |
| $\text{log}$ NLR² | 0.79 (0.54–1.15) | 0.225 | 0.81 (0.55–1.21) | 0.302 |
| White blood cell count | 1.03 (0.96–1.11) | 0.401 | 1.02 (0.95–1.10) | 0.545 |
| **IPW Distribution (**$\text{n}\text{=545}$**)** | **Mean ± SD** | **Median (Q1–Q3)** | **Range** |  |
|  | 1.01 ± 0.43 | 0.85 (0.74–1.11) | 0.62–4.32 |  |

*IPW, inverse probability weighting; OR, odds ratio; CI, confidence interval; SD, standard deviation; Q1, first quartile; Q3, third quartile; NLR, neutrophil-to-lymphocyte ratio.*

*¹ CRP was natural log-transformed using log(x+1).*

*² NLR was natural log-transformed using log(x+1).*

*Stabilized inverse probability weights were derived from a logistic regression model for CT completion. This table presents a supplementary comparison of unweighted and IPW-weighted regression estimates using covariates from the CT-completion model and should be interpreted separately from the final multivariable model.*

## Table S4c. Covariate balance before and after inverse probability weighting

| **Covariate** | **SMD (Unweighted)** | **SMD (Weighted)** |
| --- | --- | --- |
| Age (years) | 0.805 | 0.024 |
| Male sex | 0.034 | 0.019 |
| Body temperature (°C) | 0.132 | 0.023 |
| CRP (mg/L) | 0.260 | 0.038 |
| NLR | 0.227 | 0.023 |
| WBC (×10⁹/L) | 0.171 | 0.007 |
| Neutrophil (%) | 0.296 | 0.036 |
| Lymphocyte (%) | 0.323 | 0.030 |
| Monocyte (%) | 0.001 | 0.036 |
| Eosinophil (%) | 0.011 | 0.026 |

*SMD, standardized mean difference. Absolute SMD < 0.1 indicates adequate balance. All post-weighting SMDs were below 0.04, confirming acceptable covariate balance after inverse probability weighting.*

### Table S5.

### A.Sensitivity analyses of detailed multivariable logistic regression models

| **Model / Variable** | **OR** | **95% CI** | **P-value** |
| --- | --- | --- | --- |
| **1. Final Log Model *(Reference model)*** |  |  |  |
| **Age (years)** | **1.02** | **1.01 – 1.03** | **<0.001** |
| Male sex | 1.53 | 1.04 – 2.24 | **0.029** |
| Temperature (°C) | 0.92 | 0.72 – 1.18 | 0.521 |
| Monocyte % | 0.94 | 0.88 – 1.01 | 0.083 |
| Eosinophil % | 0.95 | 0.85 – 1.06 | 0.374 |
| log CRP | 1.42 | 1.18 – 1.70 | **<0.001** |
| **log LMR** | **1.86** | **1.10 – 3.15** | **0.021** |
| **2. Categorical Cutoffs Model** *(Clinical version)* |  |  |  |
| Age 65–74 years | 1.67 | 1.08 – 2.60 | **0.022** |
| Age ≥75 years | 2.07 | 1.31 – 3.27 | **0.002** |
| Male sex | 1.40 | 0.96 – 2.03 | 0.082 |
| Temperature 37.3–<38.0°C | 0.70 | 0.43 – 1.14 | 0.152 |
| Temperature ≥38.0°C | 0.76 | 0.46 – 1.25 | 0.283 |
| WBC 4–10 ×10⁹/L | 0.67 | 0.39 – 1.17 | 0.159 |
| WBC >10 ×10⁹/L | 0.92 | 0.39 – 2.19 | 0.854 |
| CRP 10–50 mg/L | 1.48 | 1.00 – 2.18 | **0.050** |
| CRP ≥50 mg/L | 3.90 | 2.00 – 7.62 | **<0.001** |
| NLR 3–5 | 0.74 | 0.45 – 1.20 | 0.220 |
| NLR ≥5 | 0.71 | 0.43 – 1.16 | 0.169 |
| **3. Raw LMR Model** *(Linear form)* |  |  |  |
| Age (years) | 1.02 | 1.01-1.03 | **<0.001** |
| Male sex | 1.43 | 0.98 – 2.08 | 0.061 |
| Temperature (°C) | 0.84 | 0.67 – 1.07 | 0.155 |
| Monocyte % | 0.93 | 0.87 – 0.99 | **0.021** |
| Eosinophil % | 0.95 | 0.86 – 1.05 | 0.344 |
| log CRP | 1.42 | 1.18 – 1.71 | **<0.001** |
| **LMR (Raw)** | **1.04** | **0.97 – 1.11** | **0.277** |
| **4. Z-score LMR Model** *(Standardized)* |  |  |  |
| Age (years) | 1.02 | 1.01-1.03 | **<0.001** |
| Male sex | 1.53 | 1.04 – 2.24 | **0.029** |
| Temperature (°C) | 0.92 | 0.72 – 1.18 | 0.521 |
| Monocyte % | 0.94 | 0.88 – 1.01 | 0.083 |
| Eosinophil % | 0.95 | 0.85 – 1.06 | 0.374 |
| log CRP | 1.42 | 1.18 – 1.70 | **<0.001** |
| **Z-score log LMR** | **1.31** | **1.04 – 1.64** | **0.021** |
| **5. LMR Tertile Model** *(Categorical grouping)* |  |  |  |
| Age (years) | 1.02 | 1.01-1.03 | **<0.001** |
| Male sex | 1.57 | 1.07 – 2.32 | **0.021** |
| Temperature (°C) | 0.94 | 0.73 – 1.21 | 0.648 |
| Monocyte % | 0.94 | 0.88 – 1.00 | 0.051 |
| Eosinophil % | 0.95 | 0.86 – 1.06 | 0.389 |
| log CRP | 1.41 | 1.17 – 1.70 | **<0.001** |
| **LMR Tertile 2 (Middle)** | **1.41** | **0.87 – 2.27** | **0.160** |
| **LMR Tertile 3 (High)** | **1.93** | **1.12 – 3.32** | **0.017** |
| **6. No LMR Model** *(Dropped LMR term)* |  |  |  |
| Age (years) | 1.02 | 1.01-1.03 | **<0.001** |
| Male sex | 1.42 | 0.98 – 2.06 | 0.065 |
| Temperature (°C) | 0.83 | 0.66 – 1.04 | 0.107 |
| **Monocyte %** | **0.91** | **0.86 – 0.97** | **0.003** |
| Eosinophil % | 0.95 | 0.86 – 1.05 | 0.326 |
| log CRP | 1.42 | 1.19 – 1.71 | **<0.001** |
| **7. IPW-Weighted Model** *(Adjusted for bias)* |  |  |  |
| Age (years) | 1.02 | 1.01-1.03 | **<0.001** |
| Male sex | 1.47 | 0.99 – 2.19 | 0.055 |
| Temperature (°C) | 0.98 | 0.76 – 1.26 | 0.853 |
| Monocyte % | 0.93 | 0.87 – 1.00 | **0.037** |
| Eosinophil % | 0.98 | 0.88 – 1.09 | 0.659 |
| log CRP | 1.42 | 1.17 – 1.73 | **<0.001** |
| **log LMR** | **1.99** | **1.13 – 3.51** | **0.017** |

*LMR, lymphocyte-to-monocyte ratio; NLR, neutrophil-to-lymphocyte ratio; CRP, C-reactive protein; WBC, white blood cell count; IPW, inverse probability weighting; OR, odds ratio; CI, confidence interval. For the clinically categorized model, the reference groups were age <65 years, female sex, temperature <37.3°C, WBC <4 x 10^9/L, CRP <10 mg/L, and NLR <3. In the tertile model, the lowest LMR tertile served as the reference category.*

### B.Sensitivity analyses of LMR transformations and age-interaction effects

| **Model** | **LMR specification** | **OR (95% CI)** | **Wald P-value** | **LRT P-value*** |
| --- | --- | --- | --- | --- |
| **Main models** |  |  |  |  |
| Final model | log_LMR | 1.86 (1.10–3.15) | **0.021** | – |
| Raw LMR model | LMR (raw) | 1.04 (0.97–1.11) | 0.277 | – |
| Z-score model | z_log_LMR | 1.31 (1.04–1.64) | **0.021** | – |
| Tertile model | T2 vs T1 | 1.41 (0.87–2.27) | 0.160 | – |
|  | T3 vs T1 | 1.93 (1.12–3.32) | **0.017** | – |
| IPW-weighted model | log_LMR | 1.99 (1.13–3.51) | **0.017** | – |
| **Interaction models** |  |  |  |  |
| Age × log_LMR | log_LMR (main) | 3.30 (0.71–15.41) | 0.128 | 0.435 |
|  | AGE × log_LMR | 0.99 (0.97–1.01) | 0.433 |  |
| Age × log_NLR | log_NLR (main) | 1.52 (0.40–5.82) | 0.537 | 0.211 |
|  | AGE × log_NLR | 0.99 (0.97–1.01) | 0.211 |  |

*LMR, lymphocyte-to-monocyte ratio; NLR, neutrophil-to-lymphocyte ratio; OR, odds ratio; CI, confidence interval; IPW, inverse probability weighting; LRT, likelihood ratio test. LRT P values compare models with and without the interaction term. Dashes indicate not applicable.*

### **Table S6. Risk stratification and clinical performance metrics**

| **Risk group** | **N** | **Events** | **Event rate**  **(%)** | **Mean predicted probability** | **Sensitivity**  **(%)** | **Specificity**  **(%)** | **PPV**  **(%)** | **NPV**  **(%)** | **Accuracy**  **(%)** |
| --- | --- | --- | --- | --- | --- | --- | --- | --- | --- |
| **Stratification results** |  |  |  |  |  |  |  |  |  |
| Low risk | 182 | 40 | 22.0 | 0.220 | – | – | – | – | – |
| Intermediate risk | 181 | 68 | 37.6 | 0.370 | – | – | – | – | – |
| High risk | 182 | 102 | 56.0 | 0.565 | – | – | – | – | – |
| **Performance metrics*** |  |  |  |  |  |  |  |  |  |
| High risk vs others |  |  |  |  | **48.6** | **76.1** | **56.0** | **70.2** | **65.5** |
| Mid-high vs low risk |  |  |  |  | **81.0** | **42.4** | **46.8** | **78.0** | **57.2** |

*PPV, positive predictive value; NPV, negative predictive value. Performance metrics were calculated using two classification rules: “high risk vs others,” in which the high-risk group was classified as test-positive, and “intermediate/high risk vs low risk,” in which the intermediate- and high-risk groups were classified as test-positive.*

### **Table S7. ROC curve analysis, Youden index, and pairwise DeLong comparisons**

| **Model** | **AUC** | **95% CI** | **Youden threshold** | **Sensitivity**  **(%)** | **Specificity**  **(%)** | **DeLong P-value*** |
| --- | --- | --- | --- | --- | --- | --- |
| CRP | 0.609 | 0.560–0.659 | 21.23 | 36.7 | 82.7 | **<0.001** |
| NLR | 0.476 | 0.426–0.526 | 1.08 | 98.1 | 4.8 | **<0.001** |
| WBC | 0.490 | 0.438–0.541 | 4.85 | 30.0 | 76.4 | **<0.001** |
| **Final model** | **0.691** | **0.645–0.736** | **0.363** | **71.4** | **61.5** | **Reference** |

*AUC, area under the receiver operating characteristic curve; CI, confidence interval; CRP, C-reactive protein; NLR, neutrophil-to-lymphocyte ratio; WBC, white blood cell count. P values were derived from DeLong tests comparing each single-marker model with the final multivariable model.*

### **Table S8. Model improvement indices (NRI and IDI)**

| **Comparison** | **NRI**  **(event)** | **NRI**  **(non-event)** | **NRI (total)** | **P value** | **IDI** | **P value** |
| --- | --- | --- | --- | --- | --- | --- |
| Final model vs CRP-only | 0.248 | 0.170 | 0.418 (0.226–0.639) | <0.001 | 0.063 (0.031–0.119) | 0.001 |
| Final model vs NLR-only | 0.267 | 0.301 | 0.568 (0.381–0.755) | <0.001 | 0.105 (0.068–0.170) | <0.001 |
| Final model vs WBC-only | 0.267 | 0.272 | 0.538 (0.383–0.746) | <0.001 | 0.102 (0.066–0.170) | <0.001 |

*NRI, net reclassification improvement; IDI, integrated discrimination improvement. NRI and IDI were calculated as continuous (category-free) measures. P values and 95% confidence intervals were obtained from 2,000 bootstrap resamples.Positive values indicate improved classification performance of the final multivariable model relative to the comparator model.*

**Table S9. Model performance metrics for predicting CT-confirmed pneumonia**

| **Metric** | **Value** | **95% CI** |
| --- | --- | --- |
| **Discrimination** |  |  |
| Apparent AUC | 0.691 | 0.645–0.736 |
| Bootstrap-corrected AUC | 0.673 | 0.630–0.716 |
| Optimism | 0.018 | – |
| **Calibration** |  |  |
| Brier score | 0.211 | – |

AUC, area under the receiver operating characteristic curve; CI, confidence interval; CT, computed tomography. Bootstrap internal validation was performed with 1,000 resamples to estimate optimism-corrected AUC. Lower Brier scores indicate better overall predictive accuracy.

**Table S10. Distribution of Respiratory Symptoms, Oxygen Saturation, and Comorbidities by Pneumonia Status in the CT-Complete Cohort (n = 545)**

| **Characteristic** | **No Pneumonia (n = 335)** | **Pneumonia (n = 210)** | **P value** |
| --- | --- | --- | --- |
| **Respiratory Symptoms** |  |  |  |
| Cough | 235 (72.3%) | 147 (70.3%) | 0.693 |
| Sputum production | 67 (20.6%) | 47 (22.5%) | 0.684 |
| Dyspnea | 11 (3.4%) | 16 (7.7%) | 0.046 |
| Hemoptysis^1^ | 0 (0.0%) | 0 (0.0%) | — |
| Chest pain | 9 (2.8%) | 1 (0.5%) | 0.114 |
| Sore throat | 76 (23.4%) | 48 (23.0%) | 0.995 |
| Upper respiratory symptoms | 14 (4.3%) | 8 (3.8%) | 0.961 |
| ≥1 respiratory symptom (cough/sputum/dyspnea) | 245 (75.4%) | 157 (75.1%) | 1.000 |
| ≥1 respiratory symptom OR SpO₂ <95% | 251 (77.2%) | 161 (77.0%) | 1.000 |
| **Oxygen Saturation** |  |  |  |
| SpO₂, % (median, IQR)^1^ | 98.0 (97.0–98.0) | 98.0 (96.0–98.0) | 0.070 |
| SpO₂ available, n | 114 | 105 |  |
| SpO₂ <95%, n/available (%) | 10/114 (8.8%) | 11/105 (10.5%) | 0.843 |
| **Comorbidities** |  |  |  |
| Hypertension | 67 (20.6%) | 62 (29.7%) | 0.023 |
| Diabetes mellitus | 29 (8.9%) | 30 (14.4%) | 0.070 |
| Coronary artery disease | 20 (6.2%) | 23 (11.0%) | 0.065 |
| Chronic lung disease | 15 (4.6%) | 15 (7.2%) | 0.288 |
| Chronic kidney disease | 6 (1.8%) | 8 (3.8%) | 0.262 |
| Malignancy | 9 (2.8%) | 15 (7.2%) | 0.029 |

*AUC, area under the receiver operating characteristic curve; CI, confidence interval; CT, computed tomography; IQR, interquartile range; SpO₂, pulse oximetry.*

^1^*Hemoptysis was reported in 0 of 534 patients; this zero frequency reflects true absence in the documented clinical notes.*

*Data are presented as n (%) unless otherwise indicated. Percentages were calculated using the number of patients with non-missing data for each variable as the denominator. P values were obtained from chi-square tests (or Fisher's exact test when expected cell counts < 5) for categorical variables and Wilcoxon rank-sum tests for continuous variables. P = 1.000 represents the exact Fisher test p-value (not a rounding artifact); respiratory symptom prevalence was virtually identical between groups. ^1^SpO₂ data were available for only 219 of 545 patients (40.2%) in the CT-complete cohort owing to the retrospective nature of data collection; results should be interpreted with caution.*

**Table S11. Sensitivity Analysis: Comparison of Model Coefficients Under Primary vs. Strict Outcome Definition (n = 544)**

| **Variable** | **Primary Outcome (CT-confirmed)** |  |  | **Strict Outcome (CT + respiratory sx/SpO₂ <95%)** |  |  |
| --- | --- | --- | --- | --- | --- | --- |
|  | **OR** | **95% CI** | **P** | **OR** | **95% CI** | **P** |
| Age (per year) | 1.020 | 1.010–1.031 | <0.001 | 1.012 | 1.001–1.022 | 0.029 |
| Male sex | 1.542 | 1.052–2.260 | 0.026 | 1.464 | 0.984–2.177 | 0.060 |
| Body temperature (°C) | 0.903 | 0.705–1.157 | 0.421 | 0.988 | 0.767–1.274 | 0.928 |
| Monocyte (%) | 0.943 | 0.883–1.006 | 0.077 | 0.957 | 0.894–1.025 | 0.213 |
| Eosinophil (%) | 0.952 | 0.853–1.062 | 0.378 | 0.980 | 0.885–1.086 | 0.702 |
| log CRP | 1.418 | 1.180–1.705 | <0.001 | 1.364 | 1.129–1.648 | 0.001 |
| log LMR | 1.838 | 1.086–3.111 | 0.023 | 1.978 | 1.166–3.354 | 0.011 |
| **Model AUC (95% CI)** | **0.693 (0.647–0.739)** |  |  | **0.668 (0.619–0.717)** |  |  |

*The primary outcome was defined as CT-confirmed pneumonia (new infiltrate verified by a treating pulmonologist). The strict outcome required CT-confirmed pneumonia PLUS at least one compatible respiratory symptom (cough, sputum production, or dyspnea) or SpO₂ <95%. Both models include all seven predictors from the final multivariable model (Table 3). One patient with missing symptom data was excluded from both models to ensure comparability (n = 544); primary-outcome estimates therefore differ slightly from those in Table 3 (n = 545). The direction and magnitude of all coefficients remain consistent across both definitions, supporting the robustness of the imaging-based outcome.*

**Table S12. Incremental Discriminative Value of Laboratory Biomarkers Beyond Respiratory Symptoms**

***Panel A. Model Discrimination (AUC Comparison, n = 534)***

| **Model** | **Variables** | **AUC (95% CI)** | **Comparison** | **DeLong P** |
| --- | --- | --- | --- | --- |
| M1 | Age + sex + temperature | 0.638 (0.590–0.686) | — | — |
| M2 | M1 + respiratory symptoms (cough, sputum, dyspnea) | 0.644 (0.596–0.692) | M2 vs M1 | 0.415 |
| M3 | M2 + monocyte% + eosinophil% + log CRP + log LMR | 0.697 (0.652–0.743) | M3 vs M2 | 0.003 |
| M3b | M1 + monocyte% + eosinophil% + log CRP + log LMR (no symptoms) | 0.695 (0.649–0.741) | M3b vs M2 | 0.009 |

***Panel B. Reclassification Metrics***

| **Comparison** | **NRI_events** | **NRI_non-events** | **NRI_total (95% CI)** | **P** | **IDI (95% CI)** | **P** |
| --- | --- | --- | --- | --- | --- | --- |
| M2 vs M1: +Respiratory symptoms | −0.789 | 0.858 | 0.069 (−0.031 to 0.174) | 0.184 | 0.007 (−0.001 to 0.015) | 0.076 |
| M3 vs M2: +All lab biomarkers | 0.120 | 0.249 | 0.369 (0.208 to 0.535) | <0.001 | 0.057 (0.038 to 0.078) | <0.001 |
| M3b vs M2: Labs vs symptoms | 0.091 | 0.169 | 0.260 | — | 0.051 | — |
| M3 vs M1: Full vs demographics | 0.129 | 0.255 | 0.385 | — | 0.064 | — |
| M3b vs M1: Labs vs demographics | 0.139 | 0.225 | 0.363 | — | 0.058 | — |

*M1 represents the minimal clinical model using only routinely available demographic and vital-sign variables. M2 adds three respiratory symptoms (cough, sputum production, dyspnea) to quantify their independent discriminative contribution. M3 further adds all four laboratory biomarkers retained by the LASSO-selected final model: monocyte percentage, eosinophil percentage, log-transformed CRP, and log-transformed LMR. M3b tests whether laboratory biomarkers alone (without symptoms) provide equivalent discrimination. DeLong P compares each model to the immediately preceding model. NRI (continuous) and IDI were calculated with 1000 bootstrap resamples; 95% CIs and P values are bootstrap-derived (shift-to-null method). Eleven patients with incomplete symptom or laboratory data were excluded (n = 534). Adding respiratory symptoms provided no significant improvement over demographics alone (M2 vs M1: ΔAUC = 0.006, P = 0.415; NRI P = 0.184). In contrast, laboratory biomarkers provided significant and clinically meaningful incremental discrimination (M3 vs M2: ΔAUC = 0.053, P = 0.003; NRI = 0.369, P < 0.001; IDI = 0.057, P < 0.001).*

## Table S13. Missing Data for Supplementary Variables

| Variable | Full Cohort (N = 902) | CT-Complete Cohort (N = 545) |
| --- | --- | --- |
| Cough | 19 (2.1%) | 11 (2.0%) |
| Sputum | 19 (2.1%) | 11 (2.0%) |
| Dyspnea | 19 (2.1%) | 11 (2.0%) |
| Hemoptysis | 19 (2.1%) | 11 (2.0%) |
| Chest pain | 19 (2.1%) | 11 (2.0%) |
| Sore throat | 19 (2.1%) | 11 (2.0%) |
| Upper respiratory symptoms | 19 (2.1%) | 11 (2.0%) |
| Fever history | 19 (2.1%) | 11 (2.0%) |
| SpO₂ | 492 (54.5%) | 326 (59.8%) |
| Hypertension | 19 (2.1%) | 11 (2.0%) |
| Diabetes | 19 (2.1%) | 11 (2.0%) |
| CAD | 19 (2.1%) | 11 (2.0%) |
| Chronic lung disease | 19 (2.1%) | 11 (2.0%) |
| Chronic kidney disease | 19 (2.1%) | 11 (2.0%) |
| Malignancy | 19 (2.1%) | 11 (2.0%) |

*Symptom and comorbidity data were retrospectively abstracted from the electronic medical record. The uniform missingness pattern (2.1% / 2.0%) reflects patients for whom chart documentation was absent or illegible. SpO₂ had substantially higher missingness (54.5% / 59.8%) because peripheral oxygen saturation was not routinely recorded for all febrile patients during the study period; SpO₂ was therefore excluded as a predictor in the incremental models (Table S12) but retained in the strict outcome definition (Table S11) to preserve the conservative intent of that sensitivity analysis.*
